# GeneDistiller—Distilling Candidate Genes from Linkage Intervals

**DOI:** 10.1371/journal.pone.0003874

**Published:** 2008-12-05

**Authors:** Dominik Seelow, Jana Marie Schwarz, Markus Schuelke

**Affiliations:** 1 Department of Neuropaediatrics, Charité University Medical School, Berlin, Germany; 2 NeuroCure Clinical Research Center, Charité University Medical School, Berlin, Germany; University of Montreal, Canada

## Abstract

**Background:**

Linkage studies often yield intervals containing several hundred positional candidate genes. Different manual or automatic approaches exist for the determination of the gene most likely to cause the disease. While the manual search is very flexible and takes advantage of the researchers' background knowledge and intuition, it may be very cumbersome to collect and study the relevant data. Automatic solutions on the other hand usually focus on certain models, remain “black boxes” and do not offer the same degree of flexibility.

**Methodology:**

We have developed a web-based application that combines the advantages of both approaches. Information from various data sources such as gene-phenotype associations, gene expression patterns and protein-protein interactions was integrated into a central database. Researchers can select which information for the genes within a candidate interval or for single genes shall be displayed. Genes can also interactively be filtered, sorted and prioritised according to criteria derived from the background knowledge and preconception of the disease under scrutiny.

**Conclusions:**

GeneDistiller provides knowledge-driven, fully interactive and intuitive access to multiple data sources. It displays maximum relevant information, while saving the user from drowning in the flood of data. A typical query takes less than two seconds, thus allowing an interactive and explorative approach to the hunt for the candidate gene.

**Access:**

GeneDistiller can be freely accessed at http://www.genedistiller.org

## Introduction

In recent years, genetic defects have been discovered for many monogenic diseases through linkage analysis, candidate gene approaches or a combination thereof. Crucial for this success were the access to large affected families in sufficient numbers or the availability of animal models that closely mimicked the human disease phenotype. However, of more than 25,000 human protein coding genes listed in the Entrez database, less than 2,000 have been associated with human disease phenotypes [1]. Geneticists are increasingly confronted with smaller families affected with rare conditions that carry “private” mutations. Nevertheless, elucidation of the gene defects in such single families has opened whole new research areas (e.g. the KE family for FOXP2 [2] in language development and the “Muscle baby” for Myostatin in muscle research [2], [3]). Linkage analyses of these small pedigrees have thus to be performed with relatively few meioses leading to more than one or to larger candidate intervals over 10 cM, whose LOD scores may remain below the threshold for significance of 3. Such large intervals may contain several hundred genes that have to be prioritised for mutation screening before labour and cost intensive gene sequencing is initiated.

**Figure 1:**
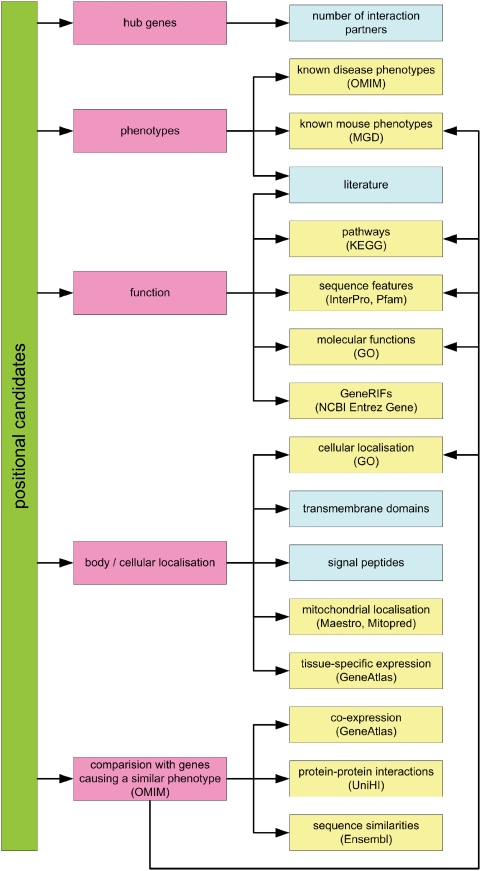
Strategies / Possibilities. This scheme illustrates different approaches to choose reasonable candidate genes from a linkage interval. The researcher can either follow a hypothesis driven approach based on a functional model or simply choose genes based on single properties reflecting the likelihood of being disease causing, e.g. the co-expression with other disease genes that cause similar phenotypes. The general concepts are depicted as pink boxes, gene properties that can be queried by GeneDistiller as yellow boxes, and properties or models GeneDistiller presently does not offer as blue boxes. With GeneDistiller, the user is absolutely free to combine gene properties according to her or his own hypotheses.

The conventional manual approach usually does not follow any strict algorithm but is guided by the background knowledge and expectations of the researcher (Figure 1). In a conventional setting, this involves a search for all known genes in the linkage interval and a subsequent query of different databases to gather available data and extract the relevant information for prioritisation. Assessment of the validity of a positional candidate requires a thorough knowledge of many data relevant to the gene or protein of interest. Most of this information can be found on the Internet, but it is tedious to collect the fragments from different data sources. While some tools offer maps showing all genes within a region (NCBI MapViewer [4], UCSC Genome Browser [5]) without any gene-specific information, others (GeneCards [6]) feature detailed genetic data but only for one single gene at a time. Besides, all these tools suffer from the lack of more elaborate query options refining the output to a well-defined group of genes.

In the past, several interactive, automatic or semiautomatic approaches to search for disease genes have been proposed [7] or implemented such as Endeavour [8], GeneWanderer [9], GeneSeeker [10], GeneSniffer (http://www.genesniffer.org/), PosMed (http://omicspace.riken.jp/PosMed/) and SUSPECTS [11]. Some applications classify genes based on sequence features [12], or use protein-protein interaction networks [9], [13] while others (GeneSeeker, SUSPECTS) combine different approaches. For the researchers, however, the algorithms of these programs remain largely inaccessible. In a meta-test of three software tools for automatic gene prioritisation of positional candidate genes the authors recommend to exert caution in relying solely on single positional candidate prioritisation tools [14]. In any case, a researcher would usually want to read relevant gene specific information for the proposed candidate genes her- or himself before embarking upon a large sequencing project.

**Figure 2:**
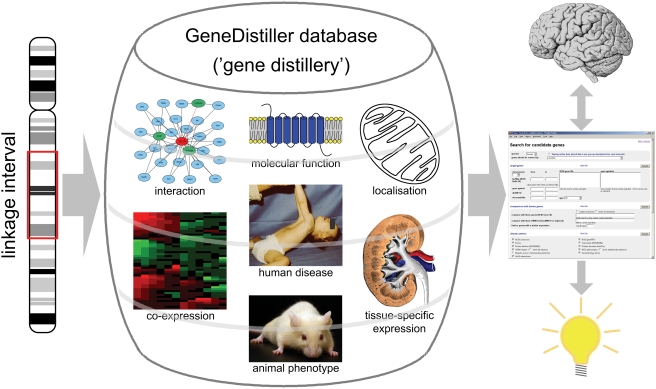
The GeneDistillery. The user-friendly interface allows the researchers to incorporate their background knowledge about diseases and genes into the interactive “gene distilling” process. They can extract all the information relevant to their specific question at our one-stop shop. This saves them from drowning in the flood of data available on the WWW and helps them to determine the most promising candidates.

GeneDistiller is aimed at various strategies. It can either be used as a tool to query, select and project genes from within a linkage interval together with gene specific data or to display rich information on human candidate genes obtained with other prioritisation tools or of the researcher's interest. Besides, it offers a customisable user-driven prioritisation integrating the available data as specified by the researcher. The application is web-based and features an intuitive interface which enables the researcher to formulate simple queries without the need to read a software manual before, yet allowing more complex queries. The software returns all results on one HTML page which can easily be printed or saved. The kind of information included is determined by the researcher. Since the results of a search are presented on the fly, the software offers a high degree of interactivity, allowing the researcher to quickly change some parameters to follow new ideas which may arise when reading the results. She can thus explore the data with the help of the computer and combine newly gained insights with her background knowledge (Figure 2).

## Results

### Strategies

GeneDistiller offers different approaches to determine the most likely candidate genes:

#### Projection

GeneDistiller can list all genes within a linkage interval together with gene specific information. Among the different kinds of gene specific data, the researcher can select those relevant to her and print and read this information for all positional candidates to choose the most promising gene. This approach can be very helpful if she has only a vague idea of the disease causing gene.

#### Selection

**Figure 3:**
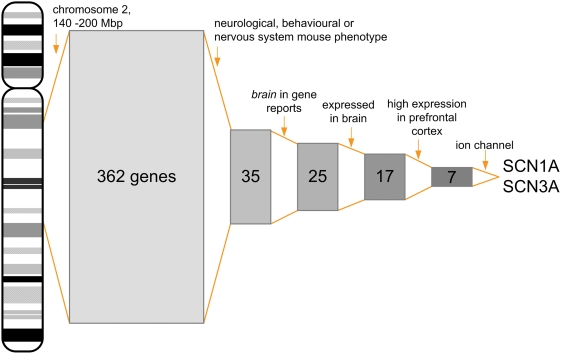
Filtering. This figure shows how filters can be applied in GeneDistiller to reduce the number of genes to be studied. After defining the linkage interval, more and more selection criteria can be added by the researcher, narrowing down the genes to ever more likely candidates. The example depicts the hunt for candidate genes for epilepsy in a 60 Mbp region on chromosome 2. The size of a rectangle is proportional to the number of genes and the grey shades reflect the “distillation” process in which the best candidates are enriched.

The researcher can apply filters to the genes in the linkage interval, thus narrowing down their number to a small group of more promising candidates (Figure 3). This approach should be applied when the researcher is able to define conditions that must be fulfilled by the candidate gene, e.g. expression in a certain tissue or co-expression with another gene. Alternatively, “visual” filters can be used to highlight gene properties so that no gene will be excluded.

#### Sorting

Genes can be sorted according to certain parameters, e.g. their position, tissue specific expression or likelihood to encode mitochondrial proteins.

#### Prioritisation

GeneDistiller offers a user-driven prioritisation function which ranks genes according to the researcher's specifications. Prioritisation approaches should be used when the researcher cannot exclude any gene in advance but wants to focus on the genes in falling order of “apparent” relevance.

The user is free to combine these methods to follow a strategy which best suits the problem, e.g. she can exclude genes using filters, choose the parameters to be used in the prioritisation process, select those to be displayed in the output and highlight interesting properties.

### Application of the different strategies

While some researchers prefer to read the available information for all genes within a candidate interval, others may rather narrow down the number of genes beforehand and focus on those fulfilling certain conditions that are regarded as mandatory. We describe the application of the two latter approaches which are more complex and most commonly used, selection (filtering) and prioritisation, here together with valid “real life” examples. More examples are given on our website and help page.

#### Selection (Figure 3)

Imagine, a candidate locus for epilepsy could be mapped to a 60 Mbp region on chromosome 2. Entering the markers limiting the interval will yield 362 genes. Since epilepsy is a common disease and a well-studied subject, the researcher might wish to focus on those genes that are known to show a suitable phenotype in an animal model. She thus filters the genes for their described mouse phenotypes. By selecting *nervous system phenotype* and *behaviour/neurological phenotype* from the *MGD phenotypes* drop-down menu and limiting the query to the respective genes, the number of genes can be significantly reduced to 35 genes which are linked to at least one of these phenotypes. A further condensation can be reached when the descriptions for human phenotypes are considered: The researcher enters the broad term *brain* into the field *highlight these keywords* and restricts the search to genes in whose descriptions one of these keywords appear. The more specific word epilepsy is not used because she does not want to restrain her search to genes already known to cause epilepsy in humans. The list now contains 25 candidate genes. Since a gene responsible for epilepsy is likely to be expressed in brain, she now opens the *expression* tab and selects *>1 (x median)* for the expression in *whole brain*. Restriction to the genes with an expression above the median can be reached when *show only genes fulfilling the conditions* is selected and will yield 17 genes. From functional studies with her patients she knows that the prefrontal cortex might be involved and decides to focus on genes with a notable expression there. Setting a filter for *prefrontal cortex expression >3 (x median)* and connecting both expression filters with *AND* shortens the list to only 7 genes. As many epilepsy genes involve ion channels she could further reduce the number of genes by adding the Gene Ontology ID for ion transport (*GO:0006811*) into the *highlight these GO IDs* fields and restrict the search to those carrying this GO ID or a subclass. Now, only 2 genes, *SCN1A* and *SCN3A* remain in the list both of which are excellent candidates for an epilepsy phenotype.

#### Prioritisation

For prioritisation the researcher can easily incorporate his or her background knowledge and follow various search avenues alone or in combination. GeneDistiller features predefined models suitable for the common approaches for prioritisation. In the output, GeneDistiller shows scores for each of the parameters chosen for the prioritisation so that the researcher can easily modify the weights given to the different parameters if she wants to shift the focus to certain aspects.

The disease under investigation may have a similar phenotype as a disease with a known gene defect or a transgenic mouse model. In this case the search comprises genes within the candidate region that are either known to be causing a similar phenotype in humans or mice or relate to disease-causing genes by experimentally proven protein-protein interactions, function in the same biochemical or signal transduction pathway, sequence similarities, similar protein domains or entries in the Gene Ontology [15] or share similar mRNA expression patterns. The latter approach stands in analogy to the credo of the neurophysiologist “*Neurons that fire together, wire together*”, changed into “*Disease-linked genes more or less, co-express*”. The set of known disease genes can either be defined by their gene symbol or gene ID or retrieved from the database by specifying suitable OMIM IDs or terms.

Genes may also be prioritised according to their suspected functional properties. The positional candidate may belong to a certain functional group of genes (e.g. various sodium channel proteins in Generalised Epilepsy with Febrile Seizures (GEFS+) [16]), biochemical pathways (e.g. O-glycolysation defects in congenital myopathies; *FCMD*, *POMGNT1*, *POMT1*, *POMT2*, *LARGE* [17]) or sub-cellular organelles (e.g. impaired mitochondrial protein synthesis in mutations of mitochondrial elongation factors *EFG1* and *TSFZ* [18], [19]).

**Figure 4:**
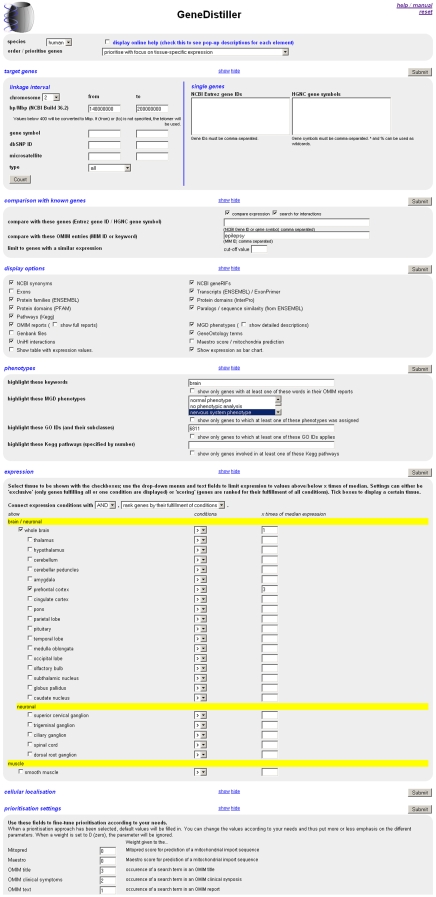
Prioritisation / query interface (screenshot). This figure shows the query interface of GeneDistiller for the prioritisation example for epilepsy described in the text. The interface is divided into different sections in which the parameters describing a similar aspect of the gene-specific data are listed. Sections not used can be closed (e.g. “prioritisation settings”). Please note that most of the available tissues in the expression section are omitted to improve readability.

**Figure 5:**
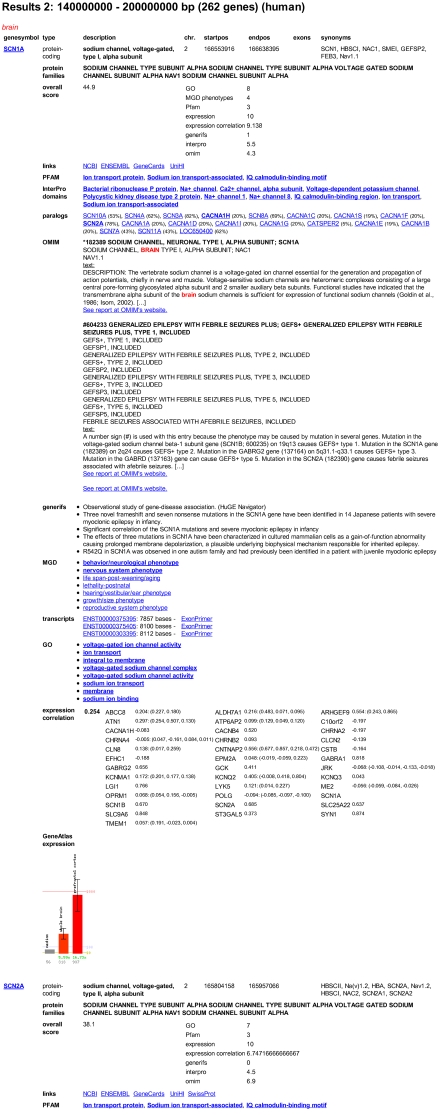
Results page (screenshot). GeneDistiller prints all results on a single HTML page. The genes are listed in the selected order, in case of prioritisation strategies also with their over-all scores and sub scores for different parameters. The gene specific data is presented with hyperlinks to the original data sources. Keywords or values that were used for filtering or highlighting are printed in bold letters. The same applies to values that are present in other genes known to be related with the selected disease (epilepsy, in this case). Please note that many NCBI GeneRIFs and OMIM reports for *SCN1A* were omitted in this figure to improve readability.

Let us imagine the same situation as depicted in the example for the selection approach with the same linkage interval. Instead of filtering the data for genes fulfilling all criteria, the genes within the linkage interval can also be prioritised according to their similarity to genes already known to cause epilepsy. Here, the predefined model *prioritise with focus on possible pathways* is chosen. We apply all our background knowledge to the query, i.e. we want to focus on genes with the assigned term *brain*, with known *nervous system phenotype* or *behaviour/neurological phenotype* in mice, expression in the relevant tissues and phenotypic, functional and expression similarities with genes known to be involved in *epilepsy* (Figure 4). As with the filtering approach, the best candidate, *SCN1A*, appears on top of the list. Here, however, no genes are discarded so that the researcher can scroll from the most to the least promising candidate, read the gene specific data and also their accordance to the prioritisation model as a whole and for every single parameter (Figure 5).

### Database schema and contents

The GeneDistiller database stores data from various sources that are most frequently considered by researchers when manually searching for candidate genes. All gene-specific data is stored in satellite tables connected to a central database table GENES in which the genes are defined. These connections are either modelled as 1∶1, 1∶n or m∶n relations, depending on the nature of the data. The database schema therefore resembles the query-optimised star schema found in many data warehouses (the database schema can be found on GeneDistiller's website). Whenever database entities are defined by a stable numeric ID in their original environment, GeneDistiller uses this ID to facilitate later updates and hyperlinks to the original source. If such an ID is not available and m∶n relations must be modelled, an internal serial numeric ID is used instead. This is also the case when data from different sources are mixed in one table, e.g. for SNP markers which are not all necessarily included in dbSNP. In the schema, SNP and STR markers are not directly connected to the genes. If marker and gene information is mixed in a query, their physical position will be used.

The database schema makes extensive use of constraints to guarantee referential integrity and to exclude worthless information (e.g. genetic markers without a position). Dubious data (e.g. markers with more than one position, except for gonosomal markers) is either excluded or this state is indicated.

GeneDistiller includes the following data: Genes, gene positions, gene RIFs, gene ontology, cellular localisation of gene products, transcripts, exons, OMIM reports, mouse phenotypes, protein-protein interactions, gene expression data, protein domains, SNP markers, STR markers. A list of the external data presently integrated in GeneDistiller is given in Table 1. More data will be added in the future according to our and the community's needs. Whenever such data is displayed, a hyperlink to the original data source is generated so that users have the chance to easily drill-down the information. Besides, links to Genbank files via BioMart [20] and to Exon-Primer (http://ihg2.helmholtz-muenchen.de/ihg/ExonPrimer.html) are presented so that the researchers can directly choose their sequencing primers without the need to manually query a sequence database.

**Table 1:** Integrated data sources.

**Genes & transcripts**	
NCBI Entrez Gene [21]	http://www.ncbi.nlm.nih.gov/sites/entrez?dbgene
ENSEMBL [22]	http://www.ensembl.org/index.html
NCBI GeneRIFs [23]	http://www.ncbi.nlm.nih.gov/sites/entrez?dbgee
**Genetic markers**	
dbSNP [24]	http://www.ncbi.nlm.nih.gov/sites/entrez?dbSnp
UniSTS [4]	http://www.ncbi.nlm.nih.gov/sites/entrez?dbunists
**Mitochondrial proteins**	
Maestro [25]	http://www.nature.com/ng/journal/v38/n5/suppinfo/ng1776_S1.html
Mitopred [26]	http://www.nature.com/ng/journal/v38/n5/suppinfo/ng1776_S1.html
**Protein domains, families and paralogs**	
ENSEMBL [22]	http://www.ensembl.org/index.html
InterPro [27]	http://www.ebi.ac.uk/interpro/
Pfam [28]	http://www.sanger.ac.uk/Software/Pfam/
**Protein functions**	
GeneOntology [15]	http://geneontology.org/
**Pathways**	
KEGG [29]	http://www.genome.jp/kegg/
**Cellular localisations**	
GeneOntology [15]	http://geneontology.org/
**Phenotypes / diseases (human)**	
OMIM [1]	http://www.ncbi.nlm.nih.gov/sites/entrez?dbOMIM
**Phenotypes (mouse)**	
MGD [30]	http://www.informatics.jax.org/
**Interactions**	
UniHI [31]	http://www.mdc-berlin.de/unihi
**Gene expression**	
GeneAtlas [32]	http://wombat.gnf.org/index.html
**External IDs**	
Swiss-Prot [33]	http://expasy.org/sprot/
UCSC [5]	http://genome.ucsc.edu/

The table lists the different data sources that are included in Gene Distiller. The data is regularly updated.

The data stored in the database is updated in regular intervals of 3 months. Further updates are performed whenever new data sources are integrated. Here again, the strict quality control measures described above are applied. Whenever data is queried, a time-stamp is printed indicating the last update or version of the data.

### Interface

GeneDistiller is web-based; all interfaces are ordinary HTML pages without any Java applications to be installed. In the query interface (Figure 4), parameters are grouped into distinct blocks. Some more advanced parameters such as tissue-specific expression are not shown by default but all blocks can be switched on and off at the researcher's will.

To use GeneDistiller, in a first step, the possible candidate genes have to be chosen. They can either be positional candidates from a linkage interval or functional candidates. In the former case, the interval can be defined by entering its position or limiting markers, in the latter case, gene symbols or Entrez gene IDs can be specified. The researcher can now select which information shall be included in the output (or stick to the default settings suitable for a first glance). Using these settings, the selected data would be shown for all genes within the candidate interval.

To reduce the number of genes, filters can be applied by specifying conditions. These can be defined either by selecting one or more values from select boxes (when only a limited number of values is stored in the respective database table, e.g. mouse phenotypes) or by typing values. Depending on the parameter, the corresponding property table is either searched for tuples with exactly this value (e.g. GO IDs) or a full-text search is performed (e.g. OMIM reports). When numeric values are stored in the database, comparison operators (<,  = , >) can be applied (e.g. for gene expression data). When the researcher does not want to exclude genes but to emphasise those fulfilling the conditions, the highlighting function can be used. In this case, keywords occurring in the text or matching values will be printed in bold and, in full-text, colour.

GeneDistiller supports the researcher with the option to sort or prioritise the genes so that the more likely candidates appear on top of the list. For sorting, a single parameter such as expression similarity or likelihood of incorporation into mitochondria can be chosen. Prioritisation offers even broader possibilities as different parameters can be combined into the ranking. The researcher can choose between different predefined settings for different prioritisation strategies (which focus on distinct approaches, e.g. similarity or tissue-specificity) and is absolutely free to choose further parameters to be included or lay more or less weight on any of them. When prioritisation is applied, a detailed prioritisation score is printed for each gene so that it becomes clear which parameter causes a gene to be highly ranked. Since a typical query takes less than 2 seconds, the researcher can easily modify his or her prioritisation settings on the basis of the results. The whole prioritisation process is therefore completely transparent and user-driven and allows a fast, intuitive, interactive and explorative access to the results.

### Output

GeneDistiller prints the results of a query in HTML format. The resulting page (Figure 5) does not make use of colour unless to highlight keywords chosen by the user. The genes are presented together with all the desired data in an order specified by the researcher and visually separated to increase readability. The page also includes hyperlinks to the original data to simplify access to more detailed data which might exist on the website of the data source. Below the actual data, a timestamp or version of the data is displayed. The page can be printed or saved for later use. The output also features two hyperlinks, one to the results page and one to the query interface with all current settings. Bookmarking this hyperlink allows a researcher to return to the query interface and change the query at any time without having to fill out the form once again. It can also be shared with other researchers so that they can refine the search on the basis of their own background knowledge or focus and eventually return their concept as another bookmarked query instead of a static list of genes.

GeneDistiller can also be called and used from other applications. Since all settings, e.g. regions, gene lists, information to be displayed filtering criteria etc., can be specified in the call, GeneDistiller can easily be integrated into other applications. This can be especially worthwhile for prioritisation tools which could extend their list of suggested candidate genes with gene specific data from GeneDistiller, hence facilitate the decision to exclude certain genes from sequencing.

## Discussion

GeneDistiller is aimed at the geneticists themselves. We have therefore developed an interface that is relatively easy to use. While this makes the use of GeneDistiller quite intuitive, queries with a high degree of complexity are not feasible. For example, filters for different kinds of data are always joined by AND. While an interface allowing to enter the Boolean logic might be useful to some bioinformaticians, we believe that it would tend to confuse the majority of geneticists.

Our software differs from the existing prioritisation tools because we deeply integrate the geneticist into the gene hunting process. In our opinion, the researcher's background knowledge and the human mind's capabilities to spontaneously associate information bear a potential that is neglected by automatic solutions. In these, the researcher can give some information about the nature of the disease before the data mining begins and exclude (negatively select) suggested candidates afterwards but he is not able to quickly apply his background knowledge in between, i.e. on the basis of the results. This is further complicated because most prioritisation tools lack the capability to display gene specific data comprehensively. Reading the rich information printed by GeneDistiller could also lead the researcher spontaneously to completely new ideas, he might thus discover something he did not expect.

However, GeneDistiller is not meant as a replacement for the existing prioritisation tools. It does for example not at all offer the same degree of sequence comparisons or evaluation of interaction networks, calculations in which computers easily outperform humans. We regard our software and automatic solutions as supplemental approaches which should be combined when a prioritisation strategy is applied. If a researcher decides to solely rely on automatic prioritisation, GeneDistiller could be a valuable resource to gather information about the candidate genes to exclude some of them before the cost-intensive sequencing process is started.

At present, GeneDistiller only offers information about human genes. We are currently integrating mouse data, as mice are often used as a model organism in gene hunting. Depending on the use of GeneDistiller by the community and suggestions from the users, other species, especially rat, might be added in the future.

## Methods

### Implementation

#### Database

The GeneDistiller database runs on PostgreSQL 8 under Debian Linux on an Intel QuadCore server with 8 GB of RAM. It uses a strictly conventional schema, no special data types or objects are used. Tables are connected with foreign keys to ensure referential integrity. The database schema is query-optimised and makes use of indexes whenever an attribute is referenced or frequently included in queries.

#### Interfaces

All database user interfaces are web-accessible using plain HTML and, for some functions such as the on-line help, JavaScript. The query interface is dynamically generated from a template, so that its elements can be created according to the database contents and to allow the form to be filled out with user settings specified in a GET or POST request. These settings can either be included in a hyperlink given together with the results or in a request made by another software when GeneDistiller's light API is used. To reduce the server's load, a static version of the query interface is created whenever data has changed and used when not called with parameters. The interfaces were developed with Firefox 2 and also tested on Internet Explorer 7 but so far, no problems with older versions or other browsers have been reported.

#### Software

The software behind the interfaces was programmed in Perl 5.8. Submitted data is read using the CGI module, HTML::Template is used to create the query interface, database connections are made with the DBI module and the DBD::Pg database driver, bar charts are created with the GD module and the Statistics::Basic::Correlation module is used to calculate Pearson correlation for expression data.

For prioritisation, the users can select among different predefined schemes for common approaches, e.g. tissue specific expression or similarity with known disease genes. If a prioritisation approach has been selected, the prioritisation section will open in the interface and the preset weights assigned to each parameter will be filled in by JavaScript. Users are absolutely free to change these settings to values that better reflect their own preconception. After the database was queried, all genes are scored according to their parameters' fulfilment of the settings made in the query interface and the weight assigned to each positive match. The genes are subsequently re-ordered by their scores.

Expression similarity is calculated using Pearson correlation. For this, the mean expression in any available tissue is used. This value can be used for prioritisation (multiplied by the user-defined weight), sorting and filtering. In the latter case, only genes with a correlation higher than the specified factor are shown.

The computation of the similarity of the user specified tissue specific expression is performed by comparison of each tissue's expression/median with the specified value. If the value is above the user input and the operator is ‘greater then’ or if it is below and ‘smaller then’ was selected, a positive score will be generated; in other cases the score will be negative. The score is calculated by division of the real expression/median by the user entered value, if the result is negative, the inverse will be taken. All scores for one gene are added to generate the final similarity score.

Querying fields with a hierarchical structure (e.g. GeneOntology) will also find descendants (subclasses) of an entity, e.g. querying for DNA repair will also find genes, which do not carry this term but its subclasses base-excision repair or mismatch repair instead. To achieve this, a recursive query is carried out using a PL/pgSQL function. Results are written into a temporary table and then used by GeneDistiller to either restrict a query or to highlight values (or their subclasses) matching the user's request.

#### API

The query interface and the results page accept input submitted as GET or POST requests and will generate and return the according HTML page. All settings which can be made in the query interface can also be included in such a call. A complete list of the options with examples is given on GeneDistiller's website. Please note that the use of the data collected in GeneDistiller might require a license.
